# Prostatic Localization of a Migrating Grass Awn Foreign Body in a Dog

**DOI:** 10.3390/vetsci7040192

**Published:** 2020-11-29

**Authors:** Maria Chiara Marchesi, Giulia Moretti, Giovanni Angeli, Francesco Birettoni, Francesco Porciello, Antonello Bufalari, Domenico Caivano

**Affiliations:** Department of Veterinary Medicine, University of Perugia, Via San Costanzo 4, 06126 Perugia, Italy; maria.marchesi@unipg.it (M.C.M.); giulia.moretti@studenti.unipg.it (G.M.); giovanni.angeli@unipg.it (G.A.); francesco.porciello@unipg.it (F.P.); antonello.bufalari@unipg.it (A.B.)

**Keywords:** canine, computed tomography, prostate, ultrasonography, vegetal foreign body

## Abstract

A 13-year-old male mixed-breed dog was examined because of hematuria and pyrexia. Ultrasonographic examination of the genitourinary tract showed the presence of a migrating grass awn in the right prostatic lobe. Laparotomy allowed, under ultrasonographic guidance, to remove entirely the migrating grass awn from the prostatic parenchyma. The recovery was uneventful and four months after the surgery the owner reported that the dog showed the complete resolution of the clinical signs and full return to normal activity. To our knowledge, this case report describes for the first time the clinical presentation, imaging findings, management and outcome for a dog with prostatic localization of a migrating grass awn.

## 1. Introduction

Migrating grass awns are a frequent cause of foreign body disease in dogs and cats during the spring and summer seasons [1,2,3,4,5]. Migratory characteristics of grass awns, attributable to their fusiform shape and backward-pointing barbs, permit an easy penetration in a unidirectional way until they lodge into a cavity or organ. The most common localizations of vegetal foreign bodies are the external ear canal, nasal cavity, interdigital spaces, conjunctiva, thoracic cavity or iliopsoas muscles [1,2,3,4,5,6]. Rarely, grass awns can migrate through the genitourinary tract and localize in the urethra, urinary bladder or vagina [2,7,8,9].

Imaging techniques such as ultrasonography, computed tomography (CT) or MRI can be useful to identify and guide the removal of grass awns that have migrated into body tissues or cavities [10,11,12,13,14,15,16]. Recently, some authors have demonstrated the usefulness of transesophageal or transthoracic ultrasonography in identifying migrating grass awns within the thorax [17,18,19]. Moreover, intraoperative ultrasonography can be useful to improve success of surgical removal of migrating grass awns within the iliopsoas muscles [20,21].

To the best of our knowledge, no reports have described the prostatic localization of migrating grass awns in dogs. Therefore, the aim of this case report is to describe the clinical presentation, imaging findings, management and outcome for a dog with a migrating grass awn in the prostate.

## 2. Case Presentation 

A 13-year-old, 15 kg, male mixed-breed dog was referred to the Veterinary Teaching Hospital of University of Perugia for a suspected foreign body in the genitourinary tract. The owner reported that the dog showed hematuria and pyrexia (40 °C) 10 days before the presentation and the referring veterinarian visualized a hyperechoic structure in the prostate during the ultrasonographic examination. The dog was treated with enrofloxacin for 10 days and an improvement of clinical signs after onset of antimicrobial treatment was observed. At presentation, the physical examination was within normal limits. All diagnostic and surgical procedures were performed after obtaining written consent from the dog’s owner. Complete blood cell count showed a mild neutrophilia and serum biochemical analysis was unremarkable.

**Figure 1:**
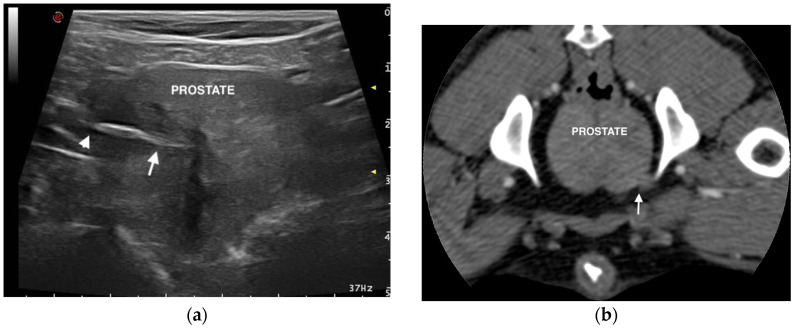
Ultrasonographic and computed tomographic (CT) images of the prostate: (**a**) Transverse ultrasonographic image of the prostate showing a spindle-shaped hyperechoic foreign body consistent with a grass awn; the tip (arrowhead) and barbs (arrow) of the awn are evident. (**b**) Transverse CT image of the prostate showing a capsular irregularity localized in the ventro-lateral right prostatic lobe, suggestive of periprostatic tissue reactions.

Ultrasonographic examination of the genitourinary tract showed the presence of a linear, spindle-shaped, hyperechoic structure of 2 cm in length localized in the prostatic parenchyma. This structure, suggestive of a migrating grass awn, was visualized in the right prostatic lobe and was surrounded by a hypoechoic/anechoic zone, consistent with a focal inflammatory response and fluid accumulation (Figure 1). The awn was orientated with the tip close to the capsule of the prostate and the barbs near the prostatic urethra.

An abdominal pre- and post-contrast CT was performed in order to identify accessory lesions in the genitourinary tract and to confirm the presence of the vegetal foreign body. CT showed no lesions secondary to the migration of the grass awn, except for the presence of irregular prostatic margins localized in the ventro-lateral right lobe (Figure 1). A mild spread of contrast was visible in this area.

After the CT, urethral and bladder endoscopy was performed with a 2.7 mm diameter fibroscope and 1.8 mm diameter working channel (Karl Storz, Germany), but no anatomical alterations and mucosal injuries were detected.

A diagnosis of prostatic migration of a grass awn was made and surgical removal of the foreign body was recommended.

**Figure 2:**
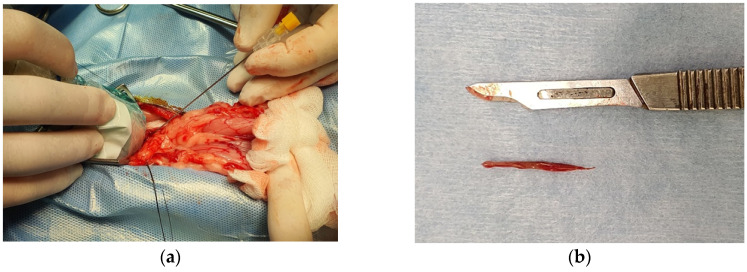
Photograph of the microconvex probe encased in a sterile protective cover and positioned on the ventral surface of the prostate during the surgery; a spinal needle was introduced, under intraoperative ultrasonographic guidance, through the prostate towards the tip of the awn (**a**). Photograph of the awn after the removal from the prostate (**b**).

The dog was premedicated with dexmedetomidine (2 µg/kg, intramuscularly) (Dextroquillan^®^, Fatro Spa, Italy) and methadone (200 µg/kg, intramuscularly) (Semfortan^®^, Dechra Veterinary Products Srl, Italy), induced with lidocaine (1.5 mg/kg, intravenously) (Lidocaina 2%^®^, Esteve Spa, Italy) and propofol (Proposure^®^, Merial Italia Spa, Italy) intravenous to effect and maintained with isoflurane (Isoflo, Esteve Spa, Italy) in 100% oxygen and sufentanil (Disufen^®^, Angenerico Spa, Italy) constant rate infusion [22,23]. The dog received preoperative carprofen (4 mg/kg, intravenously) (Rimadyl^®^, Zoetis, Italy) and postoperative buprenorphine (10 µg/kg, intramuscularly) (Buprenodale^®^, Dechra Veterinary Product Srl, Italy). Preoperative abdominal ultrasonography was also performed with the dog under general anesthesia to visualize the position of the migrating grass awn in the prostate. After the scrub, the dog was placed in dorsal recumbency at the operating table, and a ventral and caudal midline celiotomy was performed through a 6 cm incision matching the location of the migrating grass awn, to allow the best-targeted approach with a minimal and effective incision. To isolate the surgical field into the abdominal cavity and to allow the intraoperative ultrasonography of the prostate, fat was gently separated by blunt dissection. The ultrasound probe (microconvex probe, MyLab 30 Vet Gold, Esaote, Italy) was encased in a sterile sheet (Delta Med Medical Device, Italy). The probe was located on the ventral surface of the prostate, just above the awn, with the marker of the probe in cranial position. Under direct ultrasonographic view of the vegetal foreign body, a 20 G × 60 mm spinal needle was introduced through the prostate towards the tip of the awn (Figure 2). A #11 blade was inserted parallel to the needle; once the blade was in position the spinal needle was removed and a 4 mm incision was made through the ventral surface of the prostate. Then, a mini-grasping instrument was gently introduced through the incision to grab and remove, under ultrasonographic guidance, the awn. A thorough ultrasonographic scan of the prostate confirmed the complete retrieval of the migrating grass awn (Figure 2). A swab was taken from both the affected regions and the vegetal foreign body was collected for microbiologic culture. The prostate incision was irrigated with warm sterile saline solution. The superficial layer of the prostate was closed with a 3-0 synthetic absorbable monofilament material in a simple interrupted pattern. The abdominal wall was finally closed in three layers, in a routine fashion. 

After 2 days of hospitalization, the dog was discharged and continued the antibiotic therapy (enrofloxacin, 5 mg/kg, once a day, orally) until bacteriological response. At the time of suture removal, abdominal ultrasonography showed an improvement of the affected prostatic lobe and no signs of peritoneal post-operative infection. Complete resolution of the clinical signs and full return to normal activity was reported from the owner four months after the surgery.

## 3. Discussion

Vegetal foreign bodies can penetrate through intact skin or natural orifices (external ear canal, nasal sinuses or oral cavity) and subsequently migrate into the body [1,2,3,4,5,6]. Migration of the grass awns through the genitourinary tract (urethra, urinary bladder or vagina) has been reported in dogs: an ascending migration through the vagina/urethra or a penetration pathway through the skin/intestinal tract has been suspected [7,8,9]. In this case report, the grass awn was visualized by ultrasonography in the right prostatic lobe with the tip localized close to the capsule of the prostate and the barbs near the prostatic urethra. This orientation of the grass awn suggested a possible penetration of the foreign body from the urethral opening, migration through the urethra and localization in the prostatic parenchyma. Moreover, no evidence of tissue reactions or fistula formations between genitourinary tract and intestinal bowels, body wall or other abdominal organs were evident. 

Abdominal ultrasonography is a safe, noninvasive and readily available imaging technique that can be useful to assess the size of the prostate and the homogenicity of its parenchyma [24]. In our case report, the ultrasonographic examination allowed the visualization of the presence of a migrating vegetal foreign body in the prostatic parenchyma. The ultrasonographic appearance of the migrating grass awn in the prostate was similar to the appearance described in various sites. A spindle shaped, hyperechoic structure, casting or not casting an acoustic shadow and frequently surrounded by a hypoechoic area are typically suggestive of migrating grass awns [3,12,14]. When a grass awn was identified by preoperative ultrasonography, intraoperative ultrasound was demonstrated to be useful to remove vegetal foreign bodies in various organs or cavities, minimizing the risk of their fragmentation or iatrogenic damage of the tissue [18,20,21]. In the dog described in this case report, intraoperative ultrasonography allowed to remove the migrating foreign body and to confirm its complete retrieval, scanning the diseased area. 

Consistent with findings in our case report, CT examination can fail to identify migrating grass awns but can be useful to visualize the tissue reactions [4,10,11]. Indeed, in our case CT showed only a rounded proliferation close to the capsule of right prostatic lobe in the ventro-lateral position, suggestive of periprostatic tissue reactions due to the presence of the grass awn.

Endoscopic examination of the lower urinary tract is a diagnostic tool frequently used in dogs with urethral and urinary bladder diseases. This allows the evaluation of the inside of organs and collection of material for further examinations [25,26,27]. The urethral mucous membrane appears physiologically with a pale rose color and a smooth surface [28]. Mucous membrane injuries of the urethra, as bleeding, erosion and ulceration, can be visualized during endoscopic examination [27,28,29]. In our case, endoscopic examination of the urethra did not detect mucous membrane injuries suggestive of migration of a grass awn, but we cannot exclude a resolution of mucosal injuries considering that the onset of clinic signs occurred 10 days before the presentation.

In the dog of this report, a surgical approach by mini-invasive ventral midline laparotomy permitted an excellent visualization of the prostate and the ultrasonographic probe could be easily positioned close to the diseased region. Moreover, the dog did not suffer post-operative complications and the clinical signs due to the migrating grass awn resolved after the surgery.

## 4. Conclusions

To the best of our knowledge, this is the first report describing ultrasonographic findings of a migrating grass awn in canine prostatic parenchyma. Migrating vegetal foreign bodies can be a diagnostic challenge for veterinarians and imaging tools, as the ultrasonographic examination, can be useful for the visualization and subsequent removal of migrating grass awns. 

## References

[1] Johnston D.E., Summers B.A. (1971). Osteomyelitis of the lumbar vertebrae in dogs caused by grass-seed foreign bodies. Aust. Vet. J..

[2] Brennan E.K., Ihrke P.J. (1983). Grass awn migration in dogs and cats: A retrospective study of 182 cases. J. Am. Vet. Med. Assoc..

[3] Frendin J., Funkquist B., Hansson K., Lönnemark M., Carlsten J. (1999). Diagnostic imaging of foreign body reactions in dogs with diffuse back pain. J. Small Anim. Pract..

[4] Schultz R.M., Zwingenberger A. (2008). Radiographic, computed tomographic, and ultrasonographic findings with migrating intrathoracic grass awns in dogs and cats. Vet. Radiol. Ultrasound.

[5] Marchesi M.C., Caivano D., Conti M.B., Beccati F., Valli L., Busechian S., Rueca F. (2019). A specific laryngeal finding in dogs with bronchial vegetal foreign bodies: A retrospective study of 63 cases. J. Vet. Med. Sci..

[6] Hicks A., Golland D., Heller J., Malik R., Combs M. (2016). Epidemiological investigation of grass seed foreign body-related disease in dogs of the Riverina District of rural Australia. Aust. Vet. J..

[7] Cherbinsky O., Westropp J., Ting S., Jones B., Pollard R. (2010). Ultrasonographic features of grass awns in the urinary bladder. Vet. Radiol. Ultrasound.

[8] Morshead D. (1983). Submucosal urethral calculus secondary to foxtail awn migration in a dog. J. Am. Vet. Med. Assoc..

[9] Agut A., Carrillo J.D., Anson A., Belda E., Soler M. (2016). Imaging diagnosis-urethrovaginal fistula caused by a migrating grass awn in the vagina. Vet. Radiol. Ultrasound.

[10] Vansteenkiste D.P., Lee K.C., Lamb C.R. (2014). Computed tomographic findings in 44 dogs and 10 cats with grass seed foreign bodies. J. Small Anim. Pract..

[11] Bouabdallah R., Moissonnier P., Delisle F., De Fornel P., Manassero M., Maaoui M., Fayolle P., Viateau V. (2014). Use of preoperative computed tomography for surgical treatment of recurrent draining tracts. J. Small Anim. Pract..

[12] Della Santa D., Rossi F., Carlucci F., Vignoli M., Kircher P. (2008). Ultrasound-guided retrieval of plant awns. Vet. Radiol. Ultrasound.

[13] Staudte K.L., Hopper B.J., Gibson N.R., Read R.A. (2004). Use of ultrasonography to facilitate surgical removal of non enteric foreign bodies in 17 dogs. J. Small Anim. Pract..

[14] Gnudi G., Volta A., Bonazzi M., Gazzola M., Bertoni G. (2005). Ultrasonographic features of grass awn migration in the dog. Vet. Radiol. Ultrasound.

[15] Whitty C., Milner H., Oram B. (2013). Use of magnetic resonance imaging in the diagnosis of spinal empyema caused by a migrating grass awn in a dog. N. Z. Vet. J..

[16] Marchegiani A., Fruganti A., Cerquetella M., Cassarani M.P., Laus F., Spaterna A. (2017). Penetrating palpebral grass awn in a dog: Unusual case of a penetrating grass awn in an eyelid. J. Ultrasound.

[17] Caivano D., Bufalari A., Giorgi M.E., Conti M.B., Marchesi M.C., Angeli G., Porciello F., Birettoni F. (2014). Imaging diagnosis—Transesophageal ultrasoundguided removal of a migrating grass awn foreign body in a dog. Vet. Radiol. Ultrasound.

[18] Caivano D., Birettoni F., Rishniw M., Bufalari A., De Monte V., Proni A., Giorgi M.E., Porciello F. (2016). Ultrasonographic findings and outcomes of dogs with suspected migrating intrathoracic grass awns: 43 cases (2010–2013). J. Am. Vet. Med. Assoc..

[19] Caivano D., Birettoni F., Marchesi M.C., Moretti G., Corda A., Petrescu V.F., Porciello F., Bufalari A. (2019). Septic Pericarditis and Cardiac Tamponade Caused by Migrating Intrathoracic Grass Awn in an English Setter Dog. Isr. J. Vet. Med..

[20] Birettoni F., Caivano D., Rishniw M., Moretti G., Porciello F., Giorgi M.E., Crovace A., Bianchini E., Bufalari A. (2017). Preoperative and intraoperative ultrasound aids removal of migrating plant material causing iliopsoas myositis via ventral midline laparotomy: A study of 22 dogs. Acta Vet. Scand..

[21] Moretti G., Birettoni F., Caivano D., Nannarone S., Crovace A., Porciello F., Bufalari A. (2019). Mini-invasive approach for removal of iliopsoas migrating grass awns with an atraumatic wound retractor. J. Small Anim. Pract..

[22] Bufalari A., Di Meo A., Nannarone S., Padua S., Adami C. (2007). Fentanyl or sufentanil continuous infusion during isoflurane anaesthesia in dogs: Clinical experiences. Vet. Res. Commun..

[23] Cerasoli I., Nannarone S., Schauvliege S., Duchateau L., Bufalari A. (2016). The effects of intravenous lidocaine before propofol induction in premedicated dogs. J. Small Anim. Pract..

[24] Gunzel-Apel A.R., Mohrke C., Poulsen Nautrup C. (2001). Colour-coded pulsed Doppler sonography of the canine testis, epididymis and prostate gland: Physiological and pathologic findings. Reprod. Domest. Anim..

[25] Cannizo K.L., McLoughlin M.A., Chew D.J., DiBartola S.P. (2001). Uroendoscopy. Evaluation of the lower urinary tract. Vet. Clin. N. Am. Small Anim. Pract..

[26] Henry C.J. (2003). Management of transitional cell carcinoma. Vet. Clin. N. Am. Small Anim. Pract..

[27] Crow S.E. (2008). Canine cancer genetics: Transitional cell carcinoma in Scottish terriers. Cancer Ther..

[28] Grzegory M., Kubiak K., Jankowski M., Spuzak J., Glińska-Suchocka K., Nicpoń J., Haloń A. (2013). Endoscopic examination of the urethra and the urinary bladder in dogs—Indications, contraindications and performance technique. Pol. J. Vet. Sci..

[29] Nikula K.L., Benjamin S.A., Angleton G.M., Lee A.C. (1989). Transitional cell carcinomas of the urinary tract in a colony of beagle dogs. Vet. Pathol..

